# Deregulation of Epigenetic Mechanisms by the Hepatitis B Virus X Protein in Hepatocarcinogenesis

**DOI:** 10.3390/v5030858

**Published:** 2013-03-18

**Authors:** Ourania M. Andrisani

**Affiliations:** Department of Basic Medical Science and Center for Cancer Research, Purdue University West Lafayette, IN 47907, USA; E-Mail: andrisao@purdue.edu; Tel.: +1-765-494-8131; Fax: +1-765-494-0781

**Keywords:** Hepatitis B virus (HBV), HBV X protein, Hepatocellular Carcinoma (HCC), HBV replication, epigenetic regulation, DNA methylation, DNA methyl transferases (DNMTs), Polycomb Repressive complex 2 (PRC2), Suz12, suppressor of zeste 12 homolog (Drosophila), Znf198, zinc finger, MYM-type 2, LSD1-Co-REST-HDAC1

## Abstract

This review focuses on the significance of deregulation of epigenetic mechanisms by the hepatitis B virus (HBV) X protein in hepatocarcinogenesis and HBV replication. Epigenetic mechanisms, DNA methylation, and specific histone modifications, e.g., trimethylation of H3 on lysine-27 or lysine-4, maintain ‘cellular memory’ by silencing expression of lineage-inducing factors in stem cells and conversely, of pluripotency factors in differentiated cells. The X protein has been reported to induce expression of DNA methyltransferases (DNMTs), likely promoting epigenetic changes during hepatocarcinogenesis. Furthermore, in cellular and animal models of X-mediated oncogenic transformation, protein levels of chromatin modifying proteins Suz12 and Znf198 are down-regulated. Suz12 is essential for the Polycomb Repressive Complex 2 (PRC2) mediating the repressive trimethylation of H3 on lysine-27 (H3K27me3). Znf198, stabilizes the LSD1-CoREST-HDAC complex that removes, via lysine demethylase1 (LSD1), the activating trimethylation of H3 on lysine-4 (H3K4me3). Down-regulation of Suz12 also occurs in liver tumors of woodchucks chronically infected by woodchuck hepatitis virus, an animal model recapitulating HBV-mediated hepatocarcinogenesis in humans. Significantly, subgroups of HBV-induced liver cancer re-express hepatoblast and fetal markers, and imprinted genes, suggesting hepatocyte reprogramming during oncogenic transformation. Lastly, down-regulation of Suz12 and Znf198 enhances HBV replication. Collectively, these observations suggest deregulation of epigenetic mechanisms by HBV X protein influences both the viral cycle and the host cell.

## 1. Introduction

Chronic hepatitis B virus (HBV) infection is a major etiologic factor in pathogenesis of hepatocellular carcinoma (HCC) [1,2]. Despite an HBV vaccine, the World Health Organization estimates 400 million people globally are chronically infected with HBV, and HCC has become the fifth most common cancer world-wide [3,4]. Liver tumors are highly heterogeneous, differing in genetic alterations, treatment, and prognosis [5]. Global transcriptome analyses of human liver tumors have identified distinct subgroups of HCCs associated with specific genetic changes and clinical prognosis [6]. In this review, of special interest is the HCC subgroup G1, identified and characterized by the transcriptome studies of Boyault *et al* [7]. The G1 subgroup is associated with low titer HBV infection, high rate of chromosomal instability, poor prognosis, and high expression levels of AFP (alpha-fetoprotein), imprinted genes (IGFII, H19, PEG3 and PEG10), and transcription factor SOX9, a key regulator of pancreatobiliary ductal system development. Given that classification of tumors by histologic markers does not identify the cellular origin of the tumor, *i.e.*, differentiated *vs.* progenitor cell, in this review I explore mechanisms that could mediate the upregulated expression of the hepatoblast/fetal markers and imprinted genes observed in the G1 subgroup of HBV-mediated HCCs. 

## 2. The HBV Life Cycle

Hepatitis B virus (HBV) is a small enveloped virus belonging to the hepadnavirus family [8]. It infects human hepatocytes causing acute and chronic liver infection and disease. Epidemiologic studies have established that chronic HBV infection, occurring in less than 5% of HBV infected patients, is associated with high risk of developing hepatocellular carcinoma by the fourth or fifth decade of life [1]. 

The HBV genome is partially double stranded and comprised of 3.2 Kb. It encodes the pre-S/S (surface antigen), the pre-C/C (core protein), the P (viral polymerase) and X open reading frames. The X protein is essential for viral transcription and replication [9,10]. Following infection of hepatocytes by the hepatitis B virus, the viral nucleocapsids are transported to the nuclear membrane where they become disassembled, releasing the viral genome into the nuclear compartment [11]. At this stage, the viral genome takes the form of relaxed, circular (RC), partially double-stranded DNA. The RC DNA is subsequently converted into covalently closed, circular DNA (cccDNA) which serves as template for transcription of the pregenomic RNA (pgRNA) and the RNA species that encode the viral proteins [12]. In turn, the pgRNA serves as the template for synthesis of the viral genome by virus-encoded reverse transcriptase [13]. This step occurs in viral capsids in the cytoplasm [14]. Importantly, the cccDNA in the nucleus of infected cells forms chromatin-like structure by association with nucleosomes [15]. Moreover, it has been clearly demonstrated that the histone modifications associated with the viral mini-chromosome determine its transcriptional activation or repression. Specifically, chromatin immunoprecipitation assays of the cccDNA/mini-chromosome have demonstrated that lysine acetylation of H3 and H4 correlates with viral replication which depends on transcription of the pgRNA, and the level of viremia in HBV-infected patients [16,17,18]. These results imply that changes in the activity of chromatin modifying complexes (*i.e.*, those that mediate epigenetic histone modifications in infected hepatocytes), influence the rate of transcription from the viral mini-chromosome, and in turn the rate of HBV replication. 

## 3. HBV and Hepatocellular Carcinoma (HCC)

Chronic infection by HBV is associated with increased risk of liver cancer development (1). Factors contributing to pathogenesis of HBV-mediated HCC include chronic liver inflammation due to continued hepatocyte death and regeneration [19]. In addition, the functions of viral proteins X [20,21], S [22] and core [23] are likely contributors to HBV-mediated hepatocarcinogenesis. In this review I focus on the role of the X protein in HBV-mediated hepatocarcinogenesis.

HBV DNA integrates into the host genome at early steps of clonal tumor expansion, the majority of tumors displaying continued expression of pX [24]. Genomic aberrations frequently occur in HBV-mediated HCC [25], but the mechanism(s) that initiate and propagate these genomic changes are not yet understood. pX is a multifunctional protein essential for the viral life cycle. Involvement of the X protein in HCC pathogenesis is supported by data from animal models revealing pX acts as a weak oncogene or a co-factor in hepatocarcinogenesis [20,21]. pX activates cellular mitogenic pathways and induces transcription of select viral and cellular genes [26,27], thereby influencing cellular proliferation mechanisms. [The reader also is referred to recent comprehensive reviews of X protein functions and HBV-mediated hepatocarcinogenesis [28,29]. 

Pertinent to the weakly oncogenic potential of the X protein, it was shown in a cellular model of immortalized hepatocytes that inducible expression of pX promotes DNA re-replication-induced DNA damage, propagation of damaged DNA to daughter cells, and generation of partial polyploidy (>4 N DNA) [30,31]. In this cellular model, it was determined pX prematurely activates the mitotic polo-like-kinase1 (Plk1) in the G2 phase [32]. Plk1 is a cell cycle regulated gene [33], required for checkpoint recovery after completion of DNA repair [34], allowing mitotic entry and cell cycle progression [35]*.* Significantly, Plk1 is over-expressed in many human cancers, including liver cancer [36], and this up-regulation correlates with poor cancer prognosis. Elevated expression of Plk1 and of a cluster of proliferation genes was also observed by microarray analyses of human HCCs, including liver tumors from chronic HBV patients [37]. Importantly, in an *in vitro* cellular model of pX-mediated hepatocyte transformation, inhibition of Plk1 suppressed transformation [31], underscoring the importance of this enzyme in HBV-induced HCC. Recent studies have linked the increased expression of Plk1 during HCC progression to down-regulation of miR-100, a microRNA that targets Plk1 [38]. Whether miR-100 becomes down-regulated in HBV-mediated HCCs remains to be determined.

Proteomic studies have shown Plk1 phosphorylates substrates involved in a wide variety of processes [39]. A classic substrate of Plk1 is the protein claspin that participates in checkpoint activation by ATR [33]; in turn, upon completion of DNA repair, the G2/M DNA damage checkpoint is terminated by proteosomal degradation of claspin [34]. This step is initiated by phosphorylation of claspin by Plk1 at phosphodegron sites, signaling ubiquitination of the substrate and proteosomal destruction [34]. The mechanism by which Plk1 induction promotes pX-mediated oncogenic transformation remains to be determined.

## 4. Chromatin Modifying Proteins Suz12 and Znf198 in the X Protein Signaling Network

A genome-wide siRNA library screen performed in pX-expressing cells led to identification of nuclear proteins Suz12 and Znf198, both components of chromatin modifying complexes, as downstream players in the pX signaling network [40]. Specifically, siRNA-mediated knockdown of these proteins rescued X-expressing cells from DNA damage induced apoptosis, suggesting a role of these proteins in X-mediated hepatocyte transformation. Loss-of-function of Znf198 and Suz12 has been described in other human cancers [41,42]. More importantly, the ZNF198 gene located on chromosome 13q12.11 is frequently affected (~30% of cases) by loss of heterozygosity in early onset HCC, correlating with high tumor grade and HBV-related tumors [43,44]. Both Suz12 [45] and Znf198 [46] associate with promyelocytic leukemia (PML) nuclear bodies (NBs) [47]; Suz12 and Znf198 knockdown cell lines exhibit reduced numbers of PML NBs [40].

PML NBs are dynamic nuclear structures that change in composition during the cell cycle [47]; are sites of epigenetic regulation [48,49], p53 function [50], DNA repair [51]; and play a role in the innate immune response [52] and viral replication [53]. Indeed, Suz12 and Znf198 knockdown cell lines, similar to PML knockdown cell lines, exhibit reduced numbers of PML NBs, loss of p53-mediated apoptosis, and loss of DNA repair [40]. Moreover, knockdown of PML, Suz12, and Znf198 proteins by siRNA transfection enhances HBV replication [40], in a cellular model of HepAD38 cells engineered to support HBV replication in a tetracycline- inducible manner. Viruses employ various mechanisms to disrupt PML function, favoring enhanced viral replication [54] For example, an early event in Herpes Simplex Virus1 (HSV1) replication, mediated by the ICP0 protein, is dislodging of HDAC1/2 from the LSD1-CoREST-HDAC complex [55,56]. In HBV replication, PML NBs become altered in both number and morphology [57]. Furthermore, HBV replication depends on modifications of H3 and H4 associated with cccDNA, the template of pgRNA transcription [16,17,18]. As described above, pgRNA transcription determines the rate of HBV replication [8], and HDAC1 associated with cccDNA correlates with decline in HBV replication [18]. 

**Figure 1:**
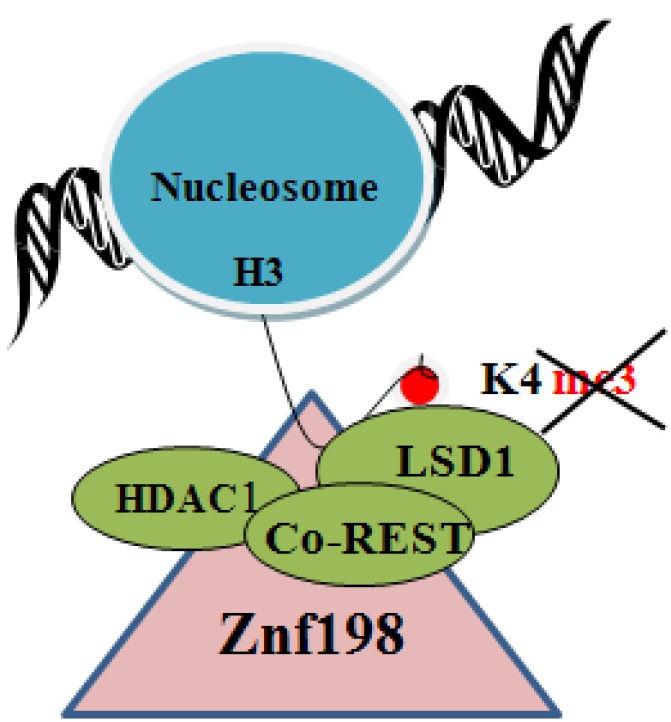
Diagram shows Znf198 interacting and stabilizing the LSD1-Co-REST-HDAC1 complex [58]. LSD1 removes the transcriptionally activating trimethylation of H3 on K4. HDAC1 removes the transcriptionally activating acetylation of histones.

Interestingly, Znf198 stabilizes the LSD1-CoREST-HDAC1 chromatin modifying complex [58] that removes histone modifications associated with transcriptional activation (Figure 1). Specifically, lysine demethylase1 (LSD1) removes the activating trimethylation of histone 3 on lysine-4 (H3K4me3), and histone deacetylase 1(HDAC1) removes the activating acetylation of histones. Thus, loss of Znf198, destabilizing the LSD1-CoREST-HDAC1 chromatin modifying complex, will maintain the transcriptionally activating histone modifications of the host chromatin as well as the viral mini-chromosome, providing yet another example of how viruses hijack cellular mechanisms to the advantage of their life cycle. 

## 5. Suz12 Containing PRC2 Complex

**Figure 2:**
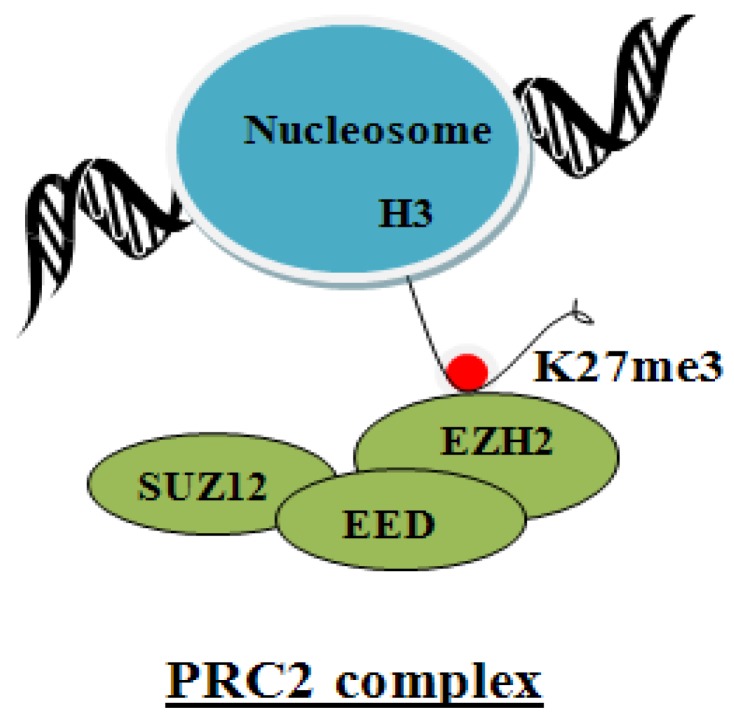
Diagram shows the essential components of the PRC2 complex, namely SUZ12, EED and EZH2. EZH2 is the histone methyltransferase enzyme that trimethylates H3 on K27. This modification silences transcription.

Suz12 is an essential component for activity of the polycomb repressive complex PRC2, comprised of histone methylstransferase EZH2 and EED [59]. The PRC2 complex (Figure 2) mediates the trimethylation of histone 3 on lysine27 (H3K27me3), a mark of transcriptionally silent chromatin [60,61]. The PRC2 chromatin modifying complex regulates expression of many developmental and signaling genes in embryonic stem cells (ESCs). Accordingly, the PRC2 complex was proposed to have a role in the maintenance of stem cell pluripotency [62,63,64,65]. However, additional studies have refined our understanding of the role of the PRC2 complex in ESCs. Specifically, PRC2-deficient ESCs failed to maintain expression of lineage-specific genes, exhibiting impaired differentiation, lack of repression of ESC markers, and lack of activation of differentiation-specific genes [66]. These observations were interpreted to mean the PRC2 complex regulates cell fate transitions during lineage commitment [66,67,68]. 

In human embryonic fibroblasts, more than 1,000 genes are transcriptionally silenced by the PRC2 complex [62]. In untransformed hepatocytes, Suz12 chromatin immunoprecipitation assays identified a subset of genes that are repressed by the PRC2 complex, including: BAMBI, CCND2, DLK1, EpCAM, and IGFII [69]. Interestingly, Suz12 occupancy at the promoters of these genes was significantly reduced in X-transformed cells, and up-regulated expression of these genes was detected in liver tumors from animal models of X-mediated and HBV-mediated hepatocarcinogenesis [69,70]. Significantly, these genes are up-regulated in the G1 subgroup of HBV-mediated HCCs [7] and also are expressed in hepatic cancer initiating/stem cells [71]. Intriguingly, these genes are also markers of normal hepatoblasts [70], raising a question of the mechanism allowing re-expression of hepatoblast markers during oncogenic transformation of differentiated hepatocytes. One might predict up-regulation of genes supporting re-establishment of the hepatoblast phenotype, resulting in lineage mis-specification and reversal of the differentiated phenotype. 

In this dynamic process of lineage change as in hepatocyte de-differentiation, it is not understood how the PRC2 complex selects genes for repression. Although several possibilities exist including interaction of the PRC2 complex with specific transcription factors, the PRC2 complex binding to specific long, noncoding RNAs [72] as well as to short RNAs transcribed from the 5' end of genes repressed by the PRC2 complex [73], suggests a likely mechanisms for target gene selection. For example, in embryonic stem cells, the PRC2 complex regulates expression of the imprinted DLK1 gene via interaction with the long noncoding RNA Gtl2 serving as cofactor [74]. Interestingly, the DLK1 gene is expressed in hepatic progenitors and hepatoblasts [70], as well as in the G1 subgroup of HBV-mediated HCCs [7]. Moreover, poor prognosis, HBV-mediated liver tumors, referred to as the C3 subgroup in the study by Toffanin *et al* [74] also re-express EpCAM and AFP, and exhibit elevated expression of a cluster of microRNAs encoded by the imprinted DLK1-DIO3 region. Taken together, both of these studies [7,74] have identified the re-expression of hepatoblast/fetal genes in the G1/C3 subgroups of HBV-mediated HCCs, significantly, genes repressed by the PRC2 complex in differentiated hepatocytes. Furthermore, these studies suggest the down-regulation of the PRC2 complex or of its activity in the G1/C3 subgroups of HBV-mediated HCCs, raising the question of how the PRC2 complex is regulated. 

## 6. Regulation of the PRC2 Complex

Studies by independent groups have identified several mechanisms by which the PRC2 complex is regulated. (1) Suz12 protein can be down-regulated via the action of microRNAs. Specifically miR-200 targets the 3'UTR of Suz12 [75]; (2) Ezh2, the component of the PRC2 complex containing the histone methyltransferase enzymatic activity, is inactivated by Akt-mediated phosphorylation [76]. Importantly, poor prognosis HBV-mediated HCCs, such as those of the G1 subgroup, exhibit enhanced activation of Akt [7]; (3) Loss of the protein JARID, enabling association of the PRC2 complex with target genes [77]; (4) Induction of the H3K27 demethylase (JMJD3 or KD6M) could also result in removal of the transcriptionally repressive H3K27me3 modification, likely resulting in re-expression of the targeted genes. Interestingly, recent studies have shown induction of JMJD3 in Epstein Barr virus infected cells and associated Hodgkin’s lymphoma [78]; (5) Lastly, an inverse relationship was noted between protein levels of Plk1 and Suz12/Znf198 in X-induced tumorigenesis [69], suggesting Suz12 and Znf198 are down-regulated via Plk1-mediated phosphorylation, promoting their proteasomal degradation. Suz12 is phosphorylated *in vivo* at a Plk1 consensus site [79] and both Suz12 and Znf198 are *in vitro* substrates of Plk1 (unpublished data). Since Plk1 is up-regulated in HBV-mediated HCCs, and the X protein activates Plk1, these chromatin modifying proteins undergo transient down-regulation during G2 and M phases of the cell cycle. The down-regulation of Suz12 and Znf198 suggests that the respective chromatin modifying complexes are concurrently inactivated. Importantly, the LSD1-CoREST-HDAC1 complex and the PRC2 complex are tethered together via the lincRNA HOTAIR, indicating that their activities are coupled, likely acting on the same gene [80]. In ESCs, genes that contain both H3K27me3 and H3K4me3 modifications are described as “poised or bivalent” genes, awaiting activation [81]. Down-regulation of these chromatin modifying complexes induces re-activation of specific genes in the respective knockdown cell lines, during progression to X-induced transformation and in animal models of X- and HBV-mediated liver cancer development [69]. 

The consequence of chronic HBV infection and continued expression of X, mediating down-regulation of these proteins through each cell cycle of an HBV infected hepatocyte, for many cell generations, is not yet understood. The gene expression program of differentiated cells is maintained after cell division by propagating the identical “chromatin landscape” of the parental cell to the daughter cell via heritable histone modifications known as epigenetic marks [82]. Recent studies in *Drosophila* have shown that propagation of the heritable chromatin landscape through cell division involves chromatin-modifying proteins including the Enhancer-of-Zeste, found to associate with newly synthesized DNA [83]. It was proposed that these histone modifying enzymes re-establish the heritable histone modifications on newly assembled unmethylated histones, serving as the epigenetic mark. Whether the PRC2 complex also serves as the epigenetic mark in mammalian cells is unknown. If that turns out to be the case, the down-regulation of Suz12 and Znf198 by pX in HBV infected hepatocytes not only alters the gene expression pattern of the hepatocyte, but its down-regulation has the potential to propagate to the subsequent cell generations an altered chromatin landscape.

## 7. Distinct Histone Modifications and DNA Methylation Landscapes in Pluripotent *vs.* Differentiated Cells

In addition to histone modifications described above, DNA methylation is another heritable modification that is linked to gene activity and maintenance of cellular memory. For example, DNA methylation at the promoter of the pluripotency factor Oct4 in a differentiated cell maintains the Oct4 gene repressed, ensuring maintenance of the differentiated state [84]. Large scale analyses examined the link between DNA methylation and histone modifications, showing that the methylation state of H3K4 is a good predictor of promoter DNA methylation. Specifically, methylation of H3K4 disrupts promoter DNA methylation by inhibiting contact between DNA methyl transferases (DNMTs) and histones [85,86]. Global comparison of the chromatin landscape between hESCs and differentiated cells [87] using Chip-Seq approaches showed the following: (1) Differentiated cells display extensive regions with repressive histone modifications (H3K27me3 and H3K9me3) in comparison to pluripotent cells; (2) In differentiated cells there is an association of H3K27me3 with promoter hypomethylation, also suggesting a connection between histone modifications and DNA methylation. Indeed, liver differentiation *in vivo* is characterized by specific demethylation in regions enriched in H3K27me3 [88]; (3) Transcribed exons in gene regions associated with H3K36me3 exhibit DNA hypermethylation [87]. Together, these results emphasize a strong connection between histone modifications and DNA methylation in determining cellular memory. Consequently, alterations in both histone modifying enzymes as well as in DNA methyltransferases (DNMTs) have the potential to alter the heritable epigenetic landscape of the cell, thereby altering its cellular memory and identity. 

## 8. DNA Methylation and the X Protein

Several studies have reported increased levels of DNMT1, DNMT3A, and DNMT3B and aberrant DNA methylation in HBV infected cells and HBV-mediated liver tumors [89,90,91]. DNMT3A and DNMT3B are *de novo* DNA methyltransferases, and DNMT1 maintains the DNA methylation pattern of the daughter strand during DNA replication, and is referred to as the maintenance methyltransferase [92]. DNMTs catalyze the addition of a methyl group to the cytosine ring of the 5'-CpG dinucleotide. 

The HBV X protein was reported to induce transcription of DNMT1, DNMT3A, and DNMT3B genes [89] and to directly interact and activate the *de novo* methyltransferase DNMT3A [90]. Specific gene promoters were shown to be methylated in HBV infected cells including the IL-4, metallothionein-1F, IGFBP3, SUFU, and TIRAP [90,93]. Moreover, these DNMTs also methylate the viral DNA, resulting in a reduction in viral replication [93,94,95]. The significance of these DNA modifications in the pathogenesis of HCC is not understood. Recent technological advances for identification of global CpG methylation patterns have been reported [96] and will undoubtedly be applied to determine the DNA methylome of HBV-mediated HCCs. It will be interesting to explore the link between pX-mediated down-regulation of the PRC2 complex and induction of DNMTs in the cellular reprogramming of HBV infected hepatocytes.

## 9. Conclusion

Since HBV infection occurs in differentiated hepatocytes, requiring the transcriptional activity of hepatocyte-specific transcription factors [97], it is intriguing that subgroups of HBV-induced HCCs express hepatoblast and fetal markers. 

The evidence presented in this review discussed the down-regulation of Suz12/PRC2 and Znf198/LSD1-CoREST-HDAC1 chromatin modifying complexes in X-mediated tumorigenesis [69] and the induction of the DNMTs in HBV-induced HCCs [89,90,91]. A third epigenetic mechanism that is likely involved in this cellular reprogramming is deregulation of microRNAs, a topic not included in this review. In the cellular context of the HBV-infected hepatocyte, it is likely that the combination of the enhanced DNMTs expression, down-regulation of the two chromatin modifying complexes and deregulated expression of microRNAs induce epigenetic reprogramming of the differentiated hepatocyte into a de-differentiated state resembling a hepatoblast/hepatic progenitor cell. This leads to re-expression of hepatoblast, fetal and imprinted genes observed in the G1 subgroup of HCCs [7] along with acquisition of enhanced proliferative potential resembling self-renewing stem cells. Based on this scenario, I propose that this combination of X-induced epigenetic processes gives rise to hepatic cancer initiating stem cells. Recent studies have established a specific molecular sequence of events mediating cellular reprogramming of somatic cells into the induced pluripotency (iPS) cells [98]. Whether epigenetic reprogramming of chronically HBV-infected hepatocytes to HCC is a random or mechanism-specific process remains to be determined. 
